# Cross-cultural adaptation, validity, and reliability of the Nepali version of the Exercise Adherence Rating Scale: a methodological study

**DOI:** 10.1186/s12955-020-01588-6

**Published:** 2020-10-07

**Authors:** Shambhu P. Adhikari, Rubee Dev, Jayana N. Shrestha

**Affiliations:** 1 Department of Physiotherapy, School of Medical Sciences, Kathmandu University, Dhulikhel, Nepal; 2 Department of Physiotherapy, Dhulikhel Hospital, Kathmandu University Hospital, Dhulikhel, Nepal; 3 School of Public Health, Sun Yat-Sen University Global Health Institute, San Yat-Sen University, Guangzhou, China

**Keywords:** Cross-cultural adaptation, Exercise adherence rating scale, Reliability, Validity

## Abstract

**Background:**

The Exercise Adherence Rating Scale (EARS) is a commonly used outcome tool, which helps to identify the adherence rate of exercises and reasons for adherence and non-adherence. There is no evidence of the availability of any measurement tools to assess exercise adherence in the Nepalese context and cultural background. Therefore, we conducted a cross-cultural adaptation of the EARS into the Nepali language and investigated its reliability and validity.

**Methods:**

Cross-cultural adaptation of the EARS was done based on Beaton guidelines. Psychometric properties were evaluated among 18 participants aged 18 years or older with pre-diabetes or confirmed diagnosis of any disease who were prescribed with home exercises by physiotherapists. Any disease that limited participants from doing exercise and individuals unwilling to participate were excluded. Reliability was evaluated through internal consistency, using Cronbach’s alpha. Exploratory Factor Analysis (EFA) was performed to explore construct validity and confirm its unidimensionality. Receiver Operating Characteristic (ROC) curve was analyzed to identify cut-off score, sensitivity and specificity of the tool.

**Results:**

The Cronbach’s alpha was 0.94 for EARS-adherence behavior. The EFA of 6-items adherence behavior revealed the presence of one factor with an eigenvalue exceeding one. The scree-plot suggested for extraction of only one factor with strong loading (75.84%). The Area Under the Curve was 0.91 with 95% confidence interval 0.77–1.00 at *p* = 0.004. The cutoff score was found 17.5 with 89% sensitivity and 78% specificity.

**Conclusions:**

The EARS was cross-culturally adapted to the Nepali language. The reliability and construct validity of the Nepali version of the EARS were acceptable to assess exercise adherence in Nepali-speaking individuals. This validated tool might facilitate the evaluation of exercise-related interventions. Future studies could investigate other psychometric properties of the Nepali EARS.

## Background

Exercise adherence is the extent to which a person’s behavior corresponds with agreed recommendations from health care providers [1]. The benefits of exercise can only be obtained when a person is adhering to the prescribed exercises. Multiple factors are associated with exercise adherence such as sociocultural factors, knowledge towards exercise, self-efficacy, ethnicity, and economic status of an individual [2]. Within physiotherapy services, the concept of exercise adherence is associated with the performance of the prescribed exercise appropriately following the advice given by the physiotherapists [3]. There is no gold standard outcome tool to measure the exercise adherence rate in the Nepalese cultural context and background. People commonly use self-reported diaries to reflect exercise adherence; however, they lack standardization, accuracy, and possess self-presentation bias that limits their validity [4]. The *Exercise Adherence Rating Scale* (EARS) is one of the commonly used outcome tools, which helps to identify the adherence rate of exercises and reasons for adherence and non-adherence [5].

The original English version of the EARS is a 16-item, self-reported questionnaire, which assesses the adherence of prescribed exercises [6]. The EARS consist of 3 sections. Section ‘A’ is about the prescribed exercise questionnaire. This section consists of 5 items, which are related to the way of doing activities and exercise that people often do to improve their physical quality of life. The section ‘A’ allows individuals to provide qualitative information about their adherence behavior. Section ‘B’ is about exercise adherence behavior, so-called exercise adherence rating scale. This section consists of 6 items, which is an actual measure to identify exercise adherence. This evaluates whether individuals do their exercise as per recommendation or not. Section ‘C’ is about reasons for adherence/non-adherence of exercises. This section consists of 10 items, which assesses factors that hinder and facilitate the exercises [5]. All the items of both section ‘B’ and ‘C’ are scored using a 5-point Likert scale (0 = completely agree to 4 = completely disagree). The positively phrased items of section ‘B’ (items 1, 4, and 6) and section ‘C’ (items 4, 5, 6, and 7) are scored reversely. The possible summed score range from 0 to 24 and 0 to 40 in section ‘B’ and ‘C’ respectively. A possible summed score of section ‘B’ and ‘C’ range from 0 to 64. A higher overall score indicates better exercise adherence [5].

The internal consistency (0.81), test–retest reliability (0.94), constructs validity (70%) and face validity of the original version of the EARS have been established [5, 6]. Acknowledged with good validity and reliability, the EARS scale has been established as an appropriate and feasible tool to assess exercise adherence.

The “cross-cultural adaptation” is a process that looks at both language (translation) and cultural adaptation issues for a questionnaire to use in another setting [7]. Cross-cultural adaptation is important when an instrument has to be used in a different language, setting and time because of the diversified context of geography, ethnicity, economic status, culture, and diseases [2, 8]. When there is no tool available to assess exercise adherence in Nepal, a tool that is valid and reliable in measuring exercise adherence of Nepali-speaking individuals was required. Following cross-cultural adaptation, it is mandatory to establish psychometric properties such as validity, reliability, sensitivity and specificity of the adopted tool to use it in the clinical practice and research. Therefore, this study aimed to conduct the cross-cultural adaptation of the EARS to the Nepali language and investigate its reliability and validity.

## Methods and materials

Permission for the cross-cultural adaptation was received from the developer of the EARS. Ethical approval was obtained from the Kathmandu University School of Medical Sciences – Institutional Review Committee (approval number: 118/19) to conduct the study. Written informed consent was obtained from all the participants before data collection.

### Cross-cultural adaptation

**Fig. 1:**
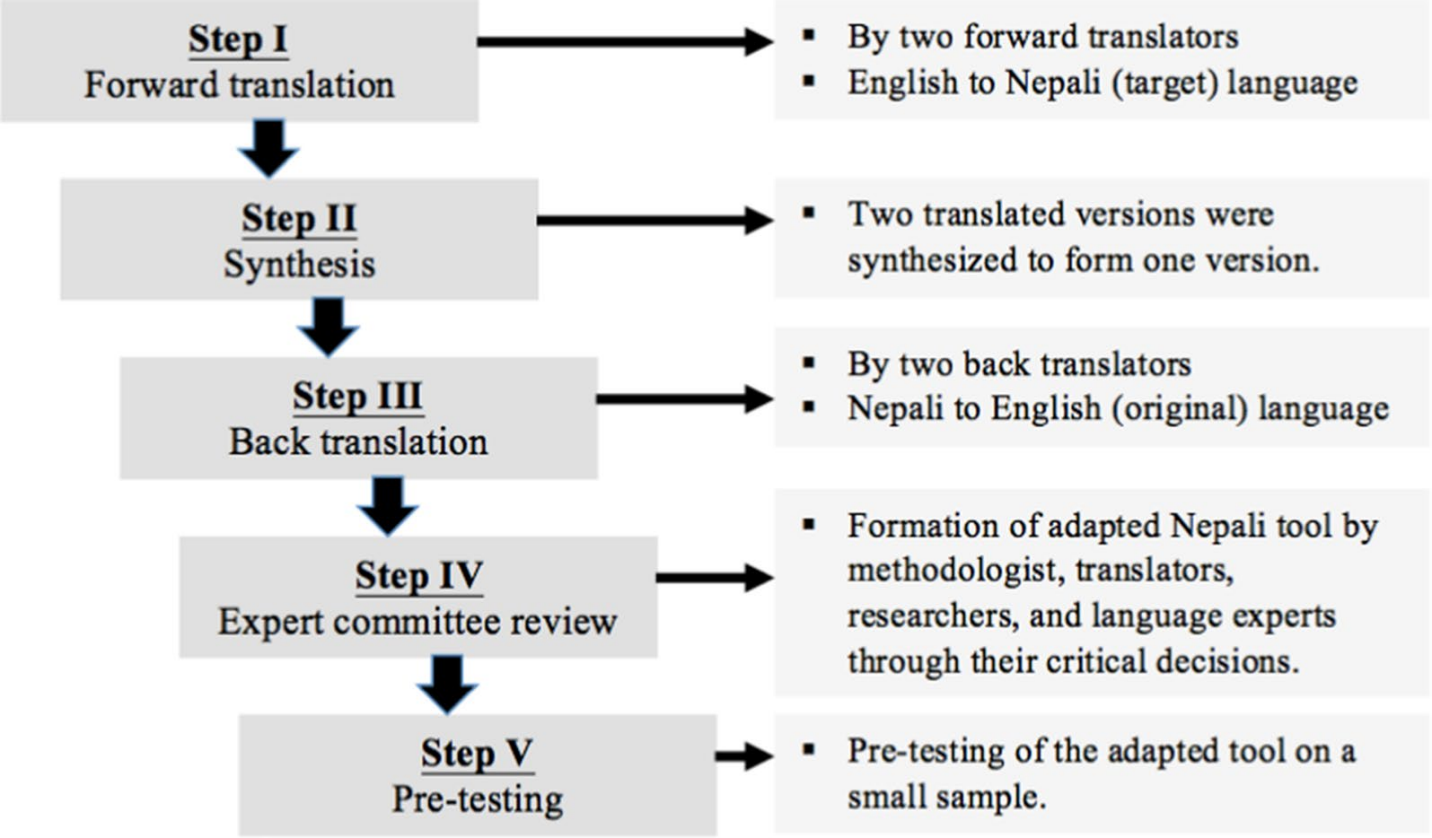
Steps of cross-cultural adaptation based on Beaton guidelines (7)

Beaton Guidelines is one of the commonly used guidelines for the translation and cross-cultural adaptation of measurement tools [7]. As per the suggestion from the developer of the tool, Dr. Emma L Godfrey, we considered (a) Beaton guidelines and (b) evidence of cross-cultural adaptation process followed in a study by Takasaki et al., in 2017 [7, 9] to cross-culturally adapt the EARS into Nepali language. The five steps of cross-cultural adaptation were; forward translation, synthesis, back translation, expert committee review, and pre-testing, which are described in Fig. 1.

Two independent non-medical translators, who were bilingual in English and Nepali language, translated the original English EARS into the Nepali language and developed two forward translated versions (FT1 and FT2). A meeting was held among two translators and investigators of the present study to reach a consensus objectively. All minor issues encountered were addressed and resolved, as there were no major issues. Through consensus, a common forward translation (FT12) was synthesized. Two Physiotherapists who were bilingual in English and Nepali language then back-translated the FT12 version into English. The purpose of the back translation was for validity checking to make sure that the adapted version was reflecting the same item content as the original version [10]. The back-translated versions were reviewed, and a consensus version was developed [7, 9].

Pretesting was done on 10 individuals with pre-diabetic conditions (who were not included in the reliability and validity testing) to explore clarity, understandability, comprehensibility, and feasibility of the adapted version of the EARS using a visual analog scale; ranging from 0 (not clear at all and difficult to understand) to 10 (clear and easy to understand). The average score of 8.1 indicated that the adapted version was clear, comprehensible, and understandable. No ambiguity of meaning on any item was reported. Thus, the pre-testing version was considered as a final version without any modification in the original English version. This was similar to the findings from De Lara et al., in which no difficulty was faced, or no suggestions were given during cross-cultural adaptation of the EARS into the Brazilian version [11]. Thus, a Nepali version of EARS (N-EARS) was cross-culturally adapted (see Additional file 1). The Nepali version of the EARS was submitted to the developer of the tool and an appreciation email response was received.

### Validation of N-EARS

Individuals at the pre-diabetic stage (who were not included during the pre-testing phase of cross-cultural adaptation) as well as patients with various conditions were screened. Patients were eligible if they satisfied the following inclusion criteria: (1) individuals with pre-diabetes (HbA1c level from 5.7 to 6.4%) [12] or any patients with a confirmed diagnosis of any disease who were prescribed with home exercises by the physiotherapists, and (2) aged 18 years or older and (3) those who were called for follow-up at the clinic after two weeks of the exercise prescription. Any disease or conditions like recent surgery that limited participants from doing exercises were excluded from the study.

### Procedure

Individuals were screened at a clinic of the Dhulikhel Hospital, Nepal from November 2019 to February 2020. During the follow-up visit after two weeks, participants’ consent was obtained, demographic information was taken, and they were asked to fill the adapted version of the EARS at the same clinic. The assessor instructed the participants who rated their level of exercise adherence and reasons for adherence/non-adherence by re-calling their exercise performance level in the previous two weeks. The individualized exercise was prescribed for 40–60 min per day for 4–6 days per week (as per common practice in Nepal) by physiotherapists. Pre-diabetic participants were purposively included in this study because all the participants were staff of the hospital and educated, which is required for any self-administered tool. Besides, we recruited participants with various conditions from the outpatient department of the hospital intending to increase the generalizability of the outcome. However, the number of participants with various conditions was small due to the literacy factor.

### Statistical analysis

The mean with standard deviation and number with percentage were described during demographic and clinical data analysis. Cronbach’s alpha (α) was calculated to determine internal consistency. Exploratory Factor Analysis (EFA) was performed in the study by Newman-Beinart et al., to determine factors in the original English tool [5]. To compare the findings with the study, we performed EFA to explore construct validity. Kaiser–Meyer–Olkin (KMO) test and Bartlett’s test were used to check for sampling adequacy and sphericity, respectively. The minimum recommended value of 0.60 was considered for sampling adequacy [13]. The Varimax rotation was used during the analysis. Eigenvalues were calculated to select the number of components in EFA [5]. Receiver Operating Characteristic (ROC) curve was analyzed to identify cutoff score, sensitivity and specificity of N-EARS. Data were analyzed using SPSS (version 21.00). The significant level was considered at *p* < 0.05.

## Results

A total of 18 individuals participated in the study. The mean age of the participants was 38 years with a standard deviation (SD) of 11.9. Two-third of the participants (n = 12, 66.6%) were females. Similarly, 12 (66.6%) participants were pre-diabetic. Out of remaining 6 (33.3%) participants, one was with anterior cruciate ligament injury of the knee (at 3 months of surgical repair), two were with low backache (one at 18 days and another at 1.5 months), one was with stroke (at 1.5 months who was able to do activities independently), one was with Bell’s palsy (at 15 days of disease onset) and one was with cardiac disease (at 3 months after open-heart surgery). Participants were from different ethnicity: Aryan (38.9%), Newar (33.3%), Mangolian (22.2%) and Tharu (5.6%), from various geographical regions: urban (38.9%), suburban (33.3%) and rural (27.8%).

As shown in Table 1, the mean score for 6-item adherence behavior and 10-item reasons for adherence/non-adherence ranged from 2.2 to 2.8 and 0.8 to 3.4 respectively. The score of item 9 of 10-item reasons for adherence/non-adherence was minimum (mean: 0.8, SD: 1.4). The exercise prescribed was mild for 10 participants (55.6%) and moderate for 8 participants (44.4%).

**Table 1:** Demographic and clinical characteristic of the participants (N = 18)

Variables	Mean (SD)	N (%)
Age (year)	38 (11.9)	
*Gender*		
Male	–	6 (33.3)
Female	–	12 (66.6)
*Participants’ conditions*		
Pre-diabetic	–	12 (66.6)
Others (ligament injury, low backache, stroke, Bell’s palsy and cardiac disease)	–	6 (33.3)
*Exercise Adherence Rating Scale*		
1. I do my exercises as often as recommended	2.8 (1.3)	–
2. I forget to do my exercises	2.5 (1.6)	–
3. I do less exercise than recommended by my health care professional	2.2 (1.4)	–
4. I fit my exercises in to my regular routine	2.6 (1.4)	–
5. I don’t get around to doing my exercises	2.8 (1.6)	–
6. I do most, or all, of my exercises	2.6 (1.3)	–
*Reasons for adherence/non-adherence*		
1. I don’t have time to do my exercises	3.0 (1.3)	–
2. Other commitments prevent me from doing my exercises	2.8 (1.4)	–
3. I don’t do my exercises when I am tired	1.6 (1.2)	–
4. I feel confident about doing my exercises	2.7 (1.2)	–
5. My family and friends encourage me to do my exercises	2.4 (1.3)	–
6. I do my exercises to improve my health	2.6 (1.4)	–
7. I do my exercises because I enjoy them	2.4 (1.5)	–
8. I adjust the way I do my exercises to suit myself	1.7 (1.6)	–
9. I stop exercising when my pain is worse	0.8 (1.4)	–
10. I’m not sure how to do my exercises	3.4 (1.3)	–

*SD* standard deviation, *N* number

### Test of reliability

As shown in Table 2, the Cronbach’s alpha was 0.94 for adherence behavior. The Cronbach’s alpha if item deleted ranged from 0.91 to 0.93 for 6-item adherence behavior. Removal of any item would result in lower Cronbach’s alpha, and therefore each item has to be retained.

**Table 2:** Internal consistency of N-EARS (N = 18)

Scale (adherence behavior)	Items	Cronbach’s alpha	Corrected item-total correlation	Cronbach’s alpha if item deleted
Exercise Adherence Rating Scale	1. I do my exercises as often as recommended	0.94	0.79	0.92
	2. I forget to do my exercises		0.71	0.93
	3. I do less exercise than recommended by my health care professional		0.71	0.93
	4. I fit my exercises in to my regular routine		0.87	0.91
	5. I don’t get around to doing my exercises		0.88	0.91
	6. I do most, or all, of my exercises		0.88	0.91

*N-EARS* Nepali-Exercise Adherence Rating Scale, *N* number

### Test of validity

Construct validity of 6-items of Section ‘B” was explored using an EFA. The KMO value for 6-items adherence behavior was 0.7, exceeding the recommended minimum value of 0.60 which verified sampling adequacy for the analysis. Bartlett’s test for sphericity indicated that correlations between items were sufficiently large (Chi-square: 110.2, *p* < 0.001) for factor analysis. Thus, the criteria for sampling adequacy and sphericity for the 6-items adherence behavior scale was achieved. As depicted in Table 3, the EFA of 6-items adherence behavior revealed the presence of one factor with an Eigen value exceeding one. The scree-plot suggested for extraction of only one factor with strong loading (75.8%).

**Table 3:** Outcome of exploratory factor analysis of 6-items of N-EARS (N = 18)

Items	Component 1	Total loading
1. I do my exercises as often as recommended	0.86	75.84%
2. I forget to do my exercises	0.78	
3. I do less exercise than recommended by my health care professional	0.79	
4. I fit my exercises in to my regular routine	0.93	
5. I don’t get around to doing my exercises	0.92	
6. I do most, or all, of my exercises	0.93	

*N-EARS* Nepali-Exercise Adherence Rating Scale, *N* number

**Fig. 2:**
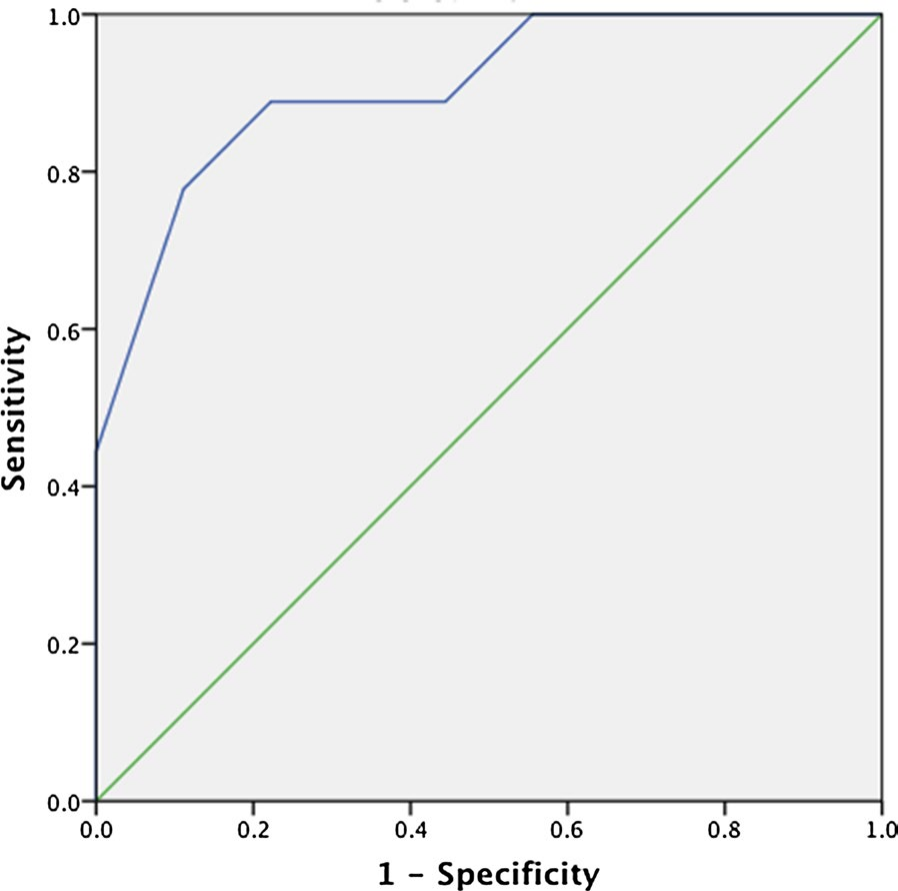
Receiver operating characteristic (ROC) curve of 6-items of adherence behavior scale

The ROC curve for the 6-items adherence behavior scale, as shown in Fig. 2, demonstrated that Area Under the Curve (AUC) was 0.9 with a 95% confidence interval 0.8–1.00 at *p* = 0.004. The cutoff score was found 17.5 with 89% sensitivity and 78% specificity.

### Correlation

As a means of validating 6-item adherence behavior, the correlation analysis was done between 6-item adherence behavior with 10-item reasons for adherence/non-adherence scale, which demonstrated significant correlation (Pearson’s Coefficient, PC: 0.8, *p* < 0.001). The 6-items adherence scale demonstrated significant correlation with item 1 (PC = 0.7, *p* = 0.002), item 2 (PC = 0.6, *p* = 0.02), item 4 (PC = 0.8, *p* < 0.001), item 6 (PC = 0.8, *p* < 0.001), item 7 (PC = 0.7, *p* = 0.001) and with item 10 (PC = 0.8, *p* < 0.001), but there was no significant correlation with items 3, 5, 8 and 9 (*p* > 0.05) of 10-item reasons for adherence/non-adherence.

## Discussion

The EARS was cross-culturally adapted to the Nepali language and the adapted N-EARS was validated. The N-EARS showed excellent internal consistency and good construct validity. The 6-items adherence behavior scale revealed the presence of only one factor with strong loading. The cutoff score was 17.5 with a sensitivity of 89% and specificity of 78%. The 6-item adherence behavior and 10-item reasons for adherence/non-adherence scale were highly correlated.

Heterogeneous participants with respect to age, gender, and diagnosis were involved in the study. The study site had easy access to the participants from urban, sub-urban as well as rural areas of Nepal. So, the participants comprised of diverse ethnicity and from various geographical regions.

### Cross-cultural adaptation

The EARS was cross-culturally adapted to the Nepali language based on Beaton guidelines [7]. The forward and back translation as well as adaptation procedure revealed no content or language-related issues. Through pre-testing, good clarity and understandability of the N-EARS were demonstrated. In contrast to the findings of a study by Meade et al., where re-framing for some items was required [6], there was no need of refining or redefining any item or words while adapting to the Nepali language. The N-EARS was formatted in such a way so that it is concise, short, easy to administer, and looks attractive. In section ‘A’ of the tool, participants did not have any issues in understanding the questions. However, in agreement with the findings from the study by Meade et al., they had difficulty in completing the answers of the questions when exercises were not prescribed in appropriate dosage or, when prescribed dosage was not understood [6].

### Reliability of N-EARS

The internal consistency was assessed to evaluate the degree of the interrelatedness among the items [14]. The internal consistency of N-EARS was excellent (α = 0.94) for 6-item adherence behavior [14, 15]. The internal consistency of the original English versions was 0.8 and that of the Brazilian version was 0.88 for 6-item adherence behavior [5, 11]. The present study demonstrated higher internal consistency (α = 0.94) of N-EARS than both English and Brazilian versions. An α value of 0.70–0.95 were considered acceptable values [16]. Therefore, the internal consistency of N-EARS of 6-item adherence behavior was comparable with the values of English as well as Brazilian versions and it was within acceptable range.

Since the recommendation was against adding up of items to calculate a final score in 10-items for reasons of adherence/non-adherence, we did not determine the internal consistency of the 10-items [5]. This was not established even in the original English version by Naomi et al. [5] and the Brazilian version by De Lira et al. [11].

### Validity of N-EARS

The EFA demonstrated adequate construct validity of the 6-item adherence behavior scale of N-EARS. The 6-item adherence scale revealed a one-factor solution with a strong loading (75.84%) to exercise adherence. The factor loading was higher than that of the original version which demonstrated 71% factor loading [5] and other self-reported outcome measures [17]. We could not perform EFA on 10-item reasons for adherence/non-adherence as it could not fulfill the criteria of sampling adequacy (KMO < 0.60) [13], which was in contrast with the Brazilian version (KMO = 0.64) [11].

The ROC curve was used to analyze the predictive effect of the 6-item adherence scale [18]. The AUC of the total score of the 6-item adherence behavior scale was 0.91 which was statistically significant and suggested a predictive validity which is in line with literature evidence [18, 19]. The cutoff score of the tool was 17.5 with a sensitivity of 89% and specificity of 78% that discriminates adherent and non-adherent participants with respect to exercises. A study by De Lira et al., in the Brazilian version demonstrated a cutoff score of 17 with sensitivity and specificity higher than 80% [11]; findings that are comparable to the present study. We also compared our findings with a study by Wang et al., in which a similar scale for exercise adherence was used. The sensitivity of 87.2% and specificity of 76.3% reported in the study was similar to the findings of our study [18].

The cutoff score of 17.5 indicated that any individual obtaining score > 17.5 out of 24 on the 6-item adherence scale is said to be adherent to the prescribed exercises. However, the cutoff score has to be cautiously used during interpretation because without knowing the level of exercise that is necessary for treatment to be effective, a cutoff score in assessing exercise adherence may not be useful [5, 19]. The cutoff score, sensitivity, and specificity reflected a preliminary predictive validity, which was not established even in the original version of the EARS and was a limitation [5]. On the other hand, completely relying on the established guidelines with the back translation reflecting the same item content as the original version supported good face validity of the N-EARS [7, 10, 20].

The correlation between the total score of 6-item adherence behavior and 10-item reasons for adherence/non-adherence demonstrated the validity of the N-EARS. The strength of correlation has been used in describing validity in patient-reported outcome measures [6, 21]. The 6-item adherence scale demonstrated a strong correlation (0.6–0.8) with items 1, 2, 4, 6, 7, and 10 of 10-item reasons for adherence/non-adherence in the present study. The reasons for adherence/non-adherence in the participants of a study by Newan-Beinart et al. were item numbers 1, 2, 3, 4, 7, and 9 [5]. Thus, the 10-items adherence/non-adherence gives clear information on reasons for adherence/non-adherence to exercise on one-to-one analysis, which may vary from one participant to another.

### Strengths and limitations

The strengths of this study include: (1) the method of cross-cultural adaptation that followed the established guidelines giving a methodological strength; (2) the reliability and validity were established on pre-diabetic who were healthy during the recruitment and on patients with various other health conditions as well. We could evaluate the feasibility of the N-EARS on healthy individuals who were recommended for exercises to prevent disease or remain fit and on patients who were prescribed exercises to treat their impairments or, activity limitations. Therefore, the reliability and validity were demonstrated in the heterogeneous group of participants; and (3) the N-EARS yielded identical psychometric properties as original EARS.

Our study also has some limitations. First, the study had a small sample size. Due to the COVID-19 pandemic, the data collection was stopped, and a preliminary analysis was done with the sample that we had collected before the study was halted in March 2020. Since preliminary analysis met the criteria for sample adequacy, the final analysis was performed with the current sample. Second, participants had to recall how much they adhered to the prescribed exercises during the last two weeks while scoring exercise adherence level. Hence, there might be a possibility of recall bias during scoring. Finally, participants had to be literate in order to respond to the EARS. This is a limitation of the EARS in a context where illiteracy is an issue. An oral response version of the scale would probably be of interest for future research.

## Conclusions

The EARS has been cross-culturally adapted to the Nepali language. This study provided excellent internal consistency and adequate face, construct as well as predictive validity of the N-EARS. The N-EARS yielded identical psychometric properties as the original EARS. A cutoff score of 17.5 was found with good sensitivity and specificity. The findings of the present study provided evidence to use N-EARS in research and clinical practice that might facilitate the evaluation of exercise-related interventions. Further studies are recommended to investigate other psychometric properties of the N-EARS with a larger sample including various diseases.

## Supplementary information

**Additional file 1.** Nepali version of Exercise Adherence Rating Scale (N-EARS).pdf.

**Additional file 2.** Dataset.

## Data Availability

The dataset supporting the conclusion of this article is available in additional files (see Additional file 2).

## Abbreviations

EARS: The Exercise Adherence Rating Scale
N-EARS: Nepali version of the Exercise Adherence Rating Scale
EFA: Exploratory Factor Analysis
ROC: Receiver Operating Characteristic
FT: Forward translation
KMO: Kaiser–Meyer–Olkin test
SD: Standard deviation
N: Number
