# Supplementary material for: Cross-cultural adaptation, validity, and reliability of the Nepali version of the Exercise Adherence Rating Scale: a methodological study
Source: Health Qual Life Outcomes. 2020 Oct 7;18:328. doi: 10.1186/s12955-020-01588-6 (PMC7538843; doi:10.1186/s12955-020-01588-6)
Supplement: Supplementary file 1 — Additional file 1. Nepali version of Exercise Adherence Rating Scale (N-EARS).pdf. [file 12955_2020_1588_MOESM1_ESM.pdf]

Exercise Adherence Rating Scale (EARS)

निर्धारित व्यायाम प्रश्नावली (खण्ड क) व्यायाम मूल्याङकन अनुपात (खण्ड ख) र तपाईलाई व्यायाम गर्न के कुराले सहयोग वा बाधा पुचाउँछ (खण्ड ग)।

खण्ड कः निर्धारित व्यायाम प्रश्नावली (Prescribed Exercise Questionnaire):

स्वास्थ्य सेवा प्रदानकर्ताहरुले सामान्यतया दिर्घरोग भएका व्यक्तिहरुलाई जीवनको गुणात्मक या आफ्नो सुधार गर्नका लागि व्यायाम र/वा अन्य क्रियाकलाप गर्न सल्लाह दिन्छन्। यस्तो व्यायाम वा क्रियाकलाप गर्न मानिसहरुले आ-आफ्नै तरिका पहिल्याँउछन्। तपाई कसरी त्यस्ता व्यायामहरु गर्नुहुन्छ हामिलाई भन्नुहोस्।

तपाईमाथि लागु हुने कोष्ठमा चिन्ह लगाउनुहोस्।

१, तपाईलाई कुन व्यायाम/क्रियाकलापहरु गर्न भनिएको छ ?<br>( ) स्वास्थ्यकर्मीसँग व्यक्तिगत अभ्यास सत्र।<br>( ) सामुहिक व्यायाम सत्र।<br>( ) स्वास्थ्यकर्मीको सिफारिश बमोजिम घरमा गर्ने<br>( ) व्यक्तिगत व्यायाम सत्र।<br>( ) हिँड्ने<br>( ) दैनिक जीवनमा सक्रिय रहने।<br>( ) अन्य	२, तपाईलाई यी व्यायाम/क्रियाकलापहरु कति पटक गर्न भनिएको छ ?<br>( ) प्रत्येक दिन<br>( ) हप्ताको ४ देखि ६ दिन<br>( ) हप्ताको २ देखि ३ दिन<br>( ) हप्ताको १ दिन<br>( ) हप्ताको १ दिन भन्दा कम<br>( ) अन्य
३, तपाईलाई यी व्यायाम/क्रियाकलापहरु कहिले सम्म गरिरहन भनिएको छ ?<br>( ) निरन्तर<br>( ) निश्चित अवधिका लागि (कृपया उल्लेख गर्नुहोस्) …….<br>……………………………………………………………<br>( ) अन्य (कृपया उल्लेख गर्नुहोस) ………………………..	४, तपाई यी व्यायाम/क्रियाकलापहरु कति पटक गर्दैहनुहुन्छ ?<br>( ) प्रत्येक दिन<br>( ) हप्ताको ४ देखि ६ दिन<br>( ) हप्ताको २ देखि ३ दिन<br>( ) हप्ताको १ दिन<br>( ) गर्दैगरेको छैन।
५, यदि तपाईले आफ्नो व्यायाम वा क्रियाकलापहरु रोक्नु भएको छ भने आफ्नो शब्दमा उल्लेख गर्नुहोस्।<br>• कहिले रोक्नुभयो? ………………………………………….<br>• किन रोक्नुभयो? ……………………………………………	६, तपाईले आफ्नो व्यायाम किन गर्नुभयो वा किन गर्नु भएन आफ्नो शब्दमा उल्लेख गर्नुहोस्।<br>• गर्नुभएको छ भने किन? ………………………………………….<br>• गर्न रोक्न भएको छ भने किन? ……………………………….

खण्ड खः व्यायाम पालना मूल्याङन अनुपात (Exercise Adherence Rating Scale - EARS):

तल दिइएको ६ वटा वाक्यहरुमा तपाई आफूलाई सल्लाह दिइएको व्यायाम कसरी गर्नु हुन्छ भन्ने कुरालाई सबै भन्दा सहि चित्रण गर्ने कोष्ठमा चिन्ह लगाउनुहोस। तपाईले दिइएको उत्तरको बारेमा सोच्दा, तपाईलाई तपाईको उपचारका निम्ति सल्लाह दिइएको व्यायामलाई ध्यान दिनुहोला।

सामाग्रीहरु	पूर्ण सहमत ०	हल्का सहमत १	तटस्थ २	हल्का असहमत ३	पूर्ण असहमत ४
१) म निर्देशन दिए बमोजिम नै व्यायाम गर्छु।					
२) म आफ्नो व्यायाम गर्न बिर्सिन्छु।					
३) स्वास्थ्यकर्मीको सल्लाह भन्दा कम व्यायाम गर्छु।					
४) म आफ्नो व्यायामलाई आफ्नो दैनिक क्रियाकलापमा मिलाउँछ।					
५) म आफ्नो व्यायाम गर्न भ्याउँदिन।					
६) म आफ्नो सबै वा धेरै जसो व्यायाम गर्छु।					

खण्ड गः तपाईलाई व्यायाम गर्न के कुराले सहयोग वा बाधा पुचाउँछ ? (What helps or hinders doing your exercises?)

सामाग्रीहरु	पूर्ण सहमत ०	हल्का सहमत १	तटस्थ २	हल्का असहमत ३	पूर्ण असहमत ४
१) मलाई व्यायाम गर्ने समय छैन ।					
२) मलाई मेरो व्यायामहरु गर्न अन्य प्रतिबध्दताले रोक्छ ।					
३) म थकित भएको बेला व्यायाम गर्दिन।					
४) म आफ्नो व्यायाम गर्ने कुरामा विश्वस्त छु ।					
५) मलाई मेरो परिवार र साथीहरुले व्यायाम गर्न प्रोत्साहान गर्दछन् ।					
६) म आफ्नो स्वास्थ्य सुधार गर्नका लागि व्यायाम गर्छु ।					
७) मलाई रमाइलो लाग्ने भएकाले व्यायाम गर्छु ।					
८) म मेरो व्यायाम मलाई सजिलो हुने किसिमले परिमार्जन गर्छु ।					
९) जब मेरो दुखाई बढ्छ तब म व्यायाम गर्दिन ।					
१०) मलाई आफ्नो व्यायाम कसरि गर्ने भन्ने थाहा छैन ।					

Scoring the exercise Adherence Rating Scale (EARS)

खण्ड ख: यो अंक दिने तरिका खण्ड ख सँग सम्बन्धित छ । EARS लाई ५ अकंको अनुपातमा मूल्याकंन गरिन्छ । (०: पूर्ण सहमत देखि ४: पूर्ण असहमत) , सामाग्री १, ४ र ६ लाई उल्टो अकं दिइनेछ । जसले ० देखि २४ अकं को रुप लिनेछ । धेरै अकंलाई व्यायाम पालना मूल्याकंन अनुपात सोही अनुसार धेरै पालना भएको मानिनेछ ।

खण्ड ग: यो अंक दिने तरिका खण्ड ग सँग सम्बन्धित छ । ५ अकंको अनुपातमा मूल्याकंन गरिन्छ । (०: पूर्ण सहमत देखि ४: पूर्ण असहमत) प्रश्न ४, ५, ६ र ७ मा उल्टो अकं दिईनुपर्ने हुन्छ । जसले गर्दा अंकले सोहि अनुसारको बढी पालना भएको जनाउनेछ । यी १० प्रश्नलाई एकै प्रश्नका रुपले पनि प्रयोग गर्न सकिनेछ जुन व्यक्तिको आवश्यक्ता अनुसार थप्न वा समावेश गर्न सकिनेछ ।

________***_______
